# Respiratory rates among rural Gambian children: a community-based cohort study

**DOI:** 10.1038/s41598-024-70796-7

**Published:** 2024-09-02

**Authors:** Polycarp Mogeni, Sharon Amima, Jennifer Gunther, Margaret Pinder, Lucy S. Tusting, Umberto D’Alessandro, Simon Cousens, Steve W. Lindsay, John Bradley

**Affiliations:** 1 Department of Infectious Disease Epidemiology, London School of Hygiene and Tropical Medicine, London, UK; 2 Kenya Medical Research Institute (KEMRI), Nairobi, Kenya; 3 Department of Food Science, Nutrition and Technology, University of Nairobi, Nairobi, Kenya; 4 Department of Biosciences, Durham University, Durham, UK; 5 Medical Research Council’s (MRC) Unit The Gambia at the London School of Hygiene and Tropical Medicine, Banjul, The Gambia; 6 Department of Disease Control, London School of Hygiene and Tropical Medicine, London, UK

**Keywords:** Respiratory rate, Centile charts, Nutrition, Ambient temperature, Health care, Medical research, Risk factors, Signs and symptoms

## Abstract

Normal respiratory rates (RR) for children under five in the tropics are well-documented, but data for older children are limited. This study tracked RR changes with age and examined associations with nutritional status and environmental factors. We monitored rural Gambian children aged 6 months to 14 years, recording RR during home visits twice weekly over two rainy seasons. Using a generalized additive model, we constructed RR reference curves, and a linear mixed-effect model identified factors influencing RR. A total of 830 children provided 67,512 RR measurements. Their median age was 6.07 years (interquartile range 4.21–8.55) and 400 (48.2%) were female. Age, stunting, ambient temperature, and time of RR measurement were independent predictors of respiratory rate. Strikingly, children showing signs of illness had greater variability in repeat RR measurements. We constructed a RR reference chart for children aged one to 13 years and proposed a cutoff of > 26 breaths/min for raised RR among children aged > 5 years bridging an important gap in this age group. Although the time of data collection, nutritional status, and ambient temperature were predictors of RR, their effect size is not clinically significant enough to warrant a change in the current WHO guidelines owing to the prevailing uncertainty in the measurement of RR. The finding that RRs between repeat measurements were more variable among children with signs of illness suggests that a single RR measurement may be inadequate to reliably assess the status of sick children—a population in which accurate diagnosis is essential to enable targeted interventions with lifesaving treatment.

## Background

Childhood respiratory illness constitutes the largest cause of post-neonatal mortality globally, a burden that could be considerably reduced by timely diagnosis and treatment^1–3^. Respiratory rate (RR) is an important vital sign monitored in clinical settings for clinical deterioration and respiratory distress and is used to support the diagnosis of severe respiratory infections^4–6^. As an indicator of respiratory function, healthcare professionals use RR to provide timely intervention, manage disease, and monitor treatment effect^6^.

The World Health Organization (WHO) defines tachypnoea (abnormally rapid breathing) as a RR ≥ 60 breaths per minute among children aged < 2 months, RR ≥ 50 among children aged between 2 and < 12 months, and RR ≥ 40 among children aged 12 months to < 60 months^4^. However, RR measurement in children is often challenging^7,8^ and although various pediatric early warning scores incorporating RR exist, such as the Pediatric Early Warning Score, there are no clear guidelines specifically tailored for children older than 5 years residing in hot, humid, and low-altitude areas in sub-Saharan Africa (sSA). RRs are typically counted manually in low-resource settings as breaths per minute using electronic timers and counting beads^9^. Though manual measurement is often the gold standard in rural sSA, it can be imprecise and subject to intra-observer variability compounded by whether the child is asleep or awake at the time when measurements are taken^8,10^. In response to calls for better pneumonia diagnosis^11,12^, automated RR counting aids are increasingly under development and evaluation^13–16^. However, there are concerns that some of these aids are out of reach or are unsuitable in resource-limited settings^8^.

RR measurements are subject to variations associated with both intrinsic factors (e.g. child age, gender, underlying health conditions, child state (asleep, awake or agitated), underlying health conditions) and extrinsic factors that include environmental and socioeconomic factors^8,17^. Younger children have higher and more variable RR measurements compared to older children whilst those residing at higher altitudes have higher RR measurements, likely due to lower oxygen concentration at higher altitudes^17–19^.

Here, we report the results of 92 consecutive rounds of community follow-up of children recruited to participate in a trial investigating the effect of improved housing on the incidence of clinical malaria in The Gambia^20^. The study was undertaken over two consecutive malaria transmission seasons between June and December of 2016 and 2017^20^. We utilized a large longitudinal dataset of RR measurements taken twice weekly to construct the RR-for-age reference curves for children aged 1–13 years and provide a comprehensive description of the predictors of RR among children residing in rural Gambia; a country at low altitude. This is one of the few studies to report RR in children aged > 5 years and the first to do so among rural Gambian children.

## Methods

### Study setting

The study was conducted in villages in the Upper River Region of The Gambia, located in the east of the country. This is an area of Sudanian savanna at an altitude of less than 50 m above sea level. The mean annual rainfall is 876 mm, occurring mainly between May and October, followed by a long dry season. The enrolled villages are located on both the north and south banks of the River Gambia; an area that experiences an average maximum daily temperature of 36 °C and a minimum of 18 °C. Further details have been reported previously^21^.

### Study design and participants

Data came from a household randomized controlled trial involving children aged 6 months to 14 years, who were recruited to a study investigating the effect of improved housing on the incidence of clinical malaria^20,21^. The study took place over a 2-year period in which clinical follow-ups were conducted over two consecutive malaria transmission seasons (June to December of 2016 and 2017)^21^. Details of the study design, data collection, consenting, supervision, and training procedures have been presented previously in a protocol^21^ and the main study results^20^. Briefly, alongside the primary trial endpoint of clinical malaria, RR data were recorded twice weekly on every participating child by trained healthcare providers equipped with electronic timers. The data were collected because of concerns that the housing intervention could cause respiratory illness due to restricting airflow through the house. The final trial results, however, showed no evidence of an increase in respiratory illness in the intervention group^20^. The assessment was done twice weekly^21^ to increase the case detection rate given the high burden of childhood pneumonia in West Africa^22^. The process involved the development of a standardized protocol containing guidelines for collecting all clinical measurements, including recording respiratory rate measurements when the child was calm. If any measurement was ≥ 50 breaths/min for children under 1 year, ≥ 40 breaths/min for children aged 1–5 years, or ≥ 25 breaths/min among children aged 5–14 years of age, a repeat measurement was taken at least 5 min after the initial measurement by the same staff member^20^. Where repeat measurements were taken, we used a second (repeat) measurement for our main analyses. Measurements were taken by observing the child’s naked chest movement, where one rise and fall of the chest was counted as one breath and recorded as the number of breaths per minute.

### Statistical analysis

#### Construction of reference curves

**Fig. 1:**
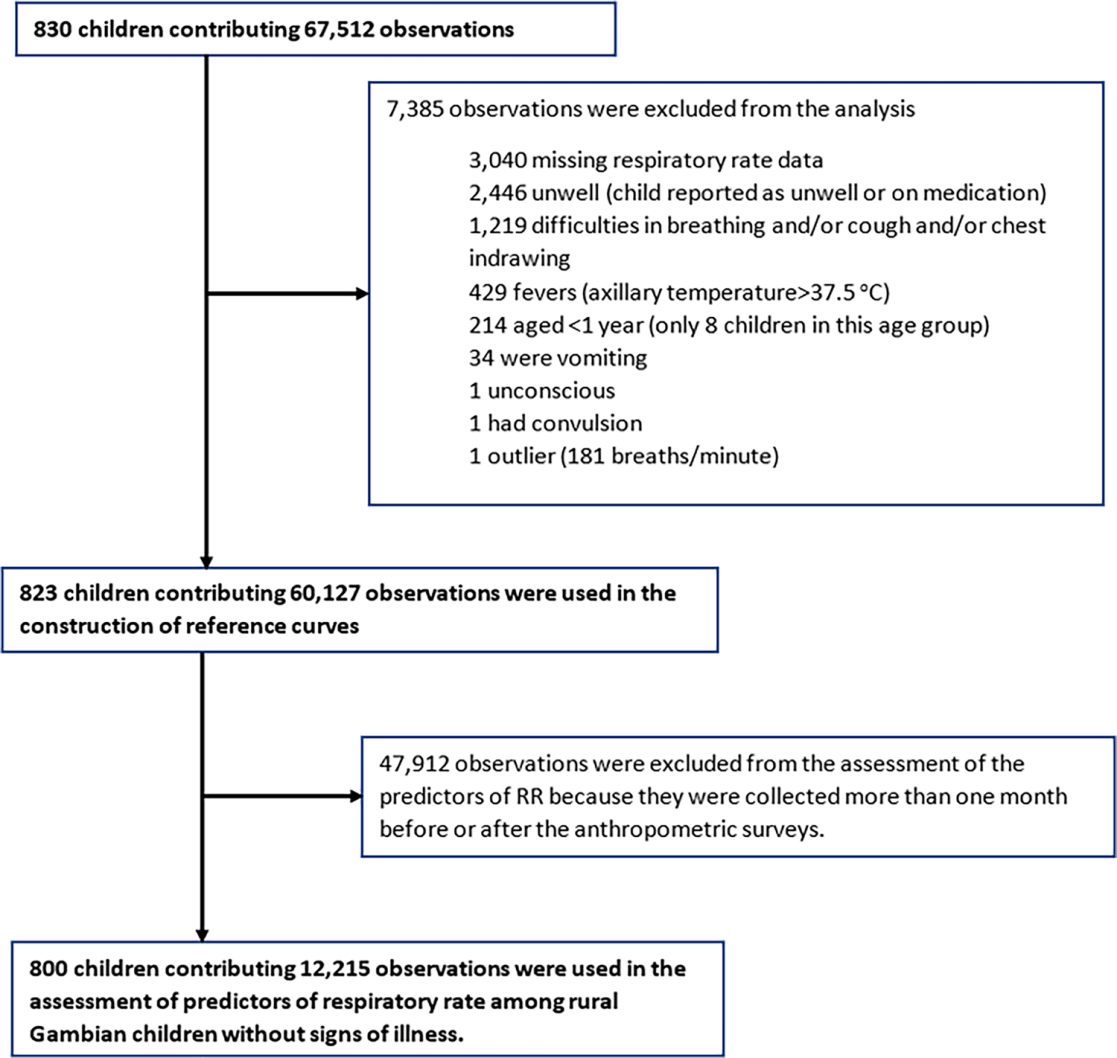
Number of participants, observations contributed, and reasons for exclusion at each analysis stage.

The main analytical objective was to construct a RR reference chart that can be used to inform clinical management of respiratory illnesses in low-altitude areas. The outcome of interest was the RR measurement derived from children who were not reported to be sick during data collection in the household. Here, we excluded data from unwell children, defined by signs of respiratory illness (cough, chest indrawing, wheezing or difficulty in breathing), fever (axillary temperature ≥ 37.5 °C), signs of gastrointestinal illness (vomiting and diarrhea), child reported as being unwell by caregiver or on medication (Fig. 1). In addition, we excluded an implausible outlier (recorded as 181 breaths/min). This measurement was not followed by a second measurement as required by the protocol^21^.

We conducted an exploratory analysis to examine potential outliers in the dataset and visually inspected the association between respiratory rate and age using the lowess function and scatter plots. Smooth percentile curves of respiratory rate as a function of age were constructed using the generalized additive models for location, scale, and shape (GAMLSS)^23^. The Box–Cox-t model^24^ was the best fit for our data among the plausible GAMLSS models (the Box–Cox-t, Box–Cox-Power-Exponential, and Box–Cox–Cole–green) that were examined in a data-driven model selection procedure^25,26^. The selected model was used to construct the reference chart (at − 2 standard deviation (SD), − 1SD, median, 1SD, and 2SD) of RR as a function of age. The results were truncated at 1 and 13 years to reduce edge effects and the impact of the limited number of study participants < 1 (n = 8) and > 13 years (n = 32) of age.

### Predictors of respiratory rate

The second analytical objective was to examine the predictors of respiratory rate to identify potential subgroups that may influence the accuracy of the constructed reference chart. We considered nutritional status, rainfall, minimum daily temperature as a proxy for ambient temperature, time of day when the measurement was taken, sex, and the randomization arm as potential predictors in the regression models. We linked the respiratory rate data to three cross-sectional anthropometric surveys conducted in December 2016, June 2017, and December 2017. The survey datasets were linked to the child’s corresponding respiratory rate observations within a month of each survey to reduce potential bias resulting from linking observations that are far apart in time. Anthropometric z-scores were calculated using the 2006 and 2007 WHO growth references for children aged < 5 years^25^ and children aged ≥ 5-years^26^ respectively. We defined stunting as a height-for-age z-score (HAZ) < − 2, underweight as weight-for-age z-score (WAZ) < − 2 and wasting as weight-for-height/length z-score (WHZ) < − 2. Daily rainfall and minimum temperatures were obtained from the meteorological department in Basse Santa Su town. We used univariable analyses (retaining covariates at *p* value < 0.1) and backward exclusion of non-significant covariates (*p* > 0.05) to arrive at a final parsimonious model. Age, sex, and randomization variables were included in multivariable models regardless of their significance in the univariable analyses. The variance inflation factor was used to examine potential collinearity.

Associations between respiratory rate and potential predictor variables were assessed using mixed-effects linear regression with random effects for each child. Age was categorized following the trend observed in the lowess curve to accommodate the non-linear trend. The likelihood ratio test statistic was used to compare a model with random intercepts only and a model containing both random intercepts and slopes. Effect estimates and their corresponding 95% confidence intervals (95% CI) were used to assess the magnitude and direction of effect for the various predictor variables included in the regression models.

In an ancillary analysis, we assessed the repeatability of RR measurements by examining the degree of agreement between repeat measurements that were taken within 5 min of each other using the Bland–Altman method of estimating the degree of agreement^27^. These repeat measurements were taken only when the first RR measurement was deemed raised for the child’s age^20^. We also examined the variations in the absolute difference between paired repeat measurements using the one-way ANOVA. Intra-observer variability was defined as the variation in repeated measurements by the same observer, and the intra-class correlation coefficient (ICC) was used to assess the reliability of these repeat measurements. The GAMLSS package^23^ in R statistical environment was used to estimate the centile curves whilst the remaining analyses were conducted using Stata 17 (Stata Corp, College Station, TX, USA).

### Role of the funding source

The funders had no role in study design, data collection, data analysis, interpretation of results, manuscript writing, or the decision to submit the manuscript for publication. The corresponding author had full access to the data and made the final decision to submit the manuscript for publication following written approval from the co-authors.

### Ethical approval

Ethical approval for the study was granted by The Gambia Government/Medical Research Council Joint Ethics Committee (reference: SCC 1390v3) and the School of Biological and Biomedical Sciences Ethics Committee, Durham University, Durham, UK (reference: SBBS/EC/1401/RooPfs). Written informed consent was obtained from the head of the household and the parent or guardian of all participating children. The study was conducted following the International Conference on Harmonization Tripartite Guideline for Good Clinical Practice and the Declaration of Helsinki.

## Results

### Reference curves

**Fig. 2:**
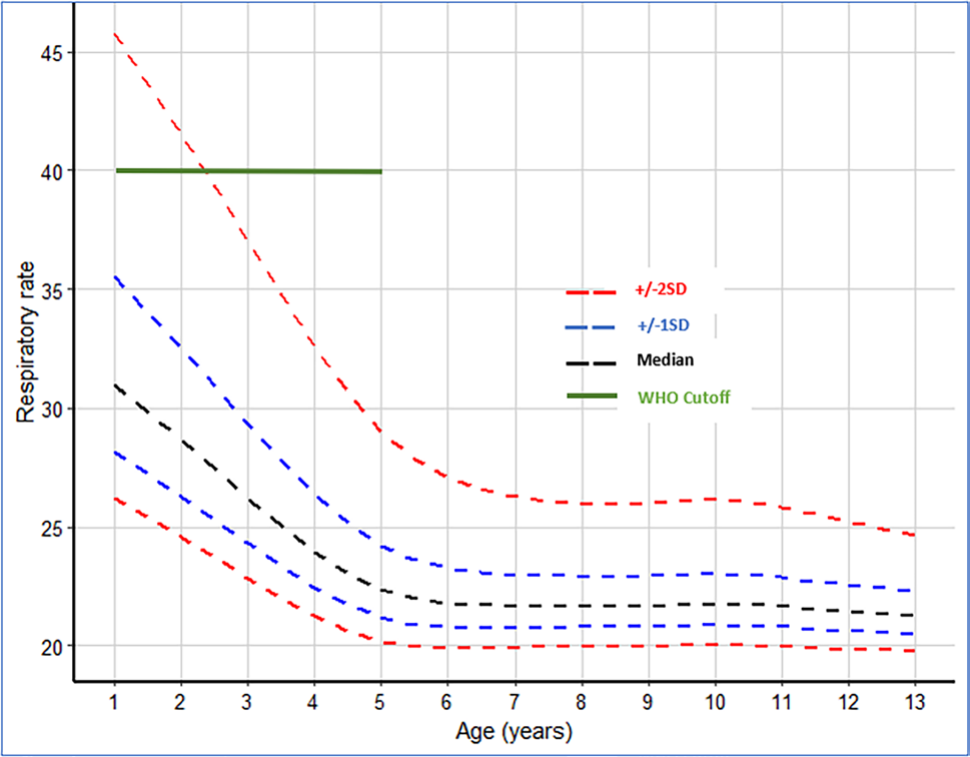
Respiratory rate (breaths/min) for age reference curves. The black dashed line represents the median RR measurements, the blue dashed lines represent the 1SD from the median, the red dashed lines represent 2SD from the median and the green continuous line represents the WHO cutoff for tachypnoea (40 breaths/min) among children aged > 12 months and < 5 years. Note that the WHO does not provide guidelines on tachypnoea among children aged above 5 years.

A total of 830 children recruited to the study contributed 67,512 observations in the analyses. The median age was 6.07 years (interquartile range (IQR), 4.21–8.55) and 400 (48.2%) were female. Details of study participants and the number of observations contributed at each stage of the analysis are presented in Fig. 1. The RR-for-age reference chart was constructed using 60,127 observations from 823 children (Fig. 2). There was a marked nonlinear decline in RR with increasing age between 1 and 6 years of age, after which the decline was minimal (the predicted median RR was 31 breaths/min [IQR 29–34] among 1-year-olds, 22 breaths/min [IQR 21–23] among 6-year-olds and 21 breaths/min [IQR 21–22] among 13-year-olds). The pre-specified cutoff for raised RR (> + 2SD) declined from 46 breaths/min among 1-year-olds to 27 breaths/min among 6-year-olds and to 25 breaths/min among 13-year-olds (Table 1, Fig. 2).

**Table 1:** Summary of children without signs of illness and their corresponding crude and model estimate of 2SD cutoff for raised respiratory rate (RR).

Age in years	Crude estimate of respiratory rate cutoff				GAMLSS estimate of RR cutoff		
	Observations	Number of children	Median (IQR)	2SD (cutoff for raised RR)	Age in Years	Median (IQR)	2SD (model cutoff for raised RR)
1 to < 1.5	410	20	32 (28–35)	41	1	31 (29–34)	46
1.5 to < 2.5	2609	89	32 (28–35)	40	2	29 (27–31)	42
2.5 to < 3.5	4308	142	30 (26–34)	38	3	26 (25–28)	37
3.5 to < 4.5	5273	177	28 (24–33)	38	4	24 (23–25)	33
4.5 to < 5.5	7180	218	25 (21–30)	38	5	22 (22–23)	29
5.5 to < 6.5	7875	237	21 (19–25)	30	6	22 (21–23)	27
6.5 to < 7.5	6753	209	20 (19–24)	29	7	22 (21–22)	26
7.5 to < 8.5	6593	193	20 (18–23)	28	8	22 (21–22)	26
8.5 to < 9.5	5821	167	20 (18–24)	28	9	22 (21–22)	26
9.5 to < 10.5	4600	142	19 (18–22)	29	10	22 (21–22)	26
10.5 to < 11.5	3881	118	20 (18–22)	28	11	22 (21–22)	26
11.5 to < 12.5	3092	92	19 (18–22)	28	12	21 (21–22)	25
12.5 to < 13.5	1478	49	19 (17–22)	26	13	21 (21–22)	25

### Respiratory rate among sick children

A total of 4119 (6.4%) observations from sick children were excluded from the construction of the reference chart. The median age of sick children was 5.5 years (IQR 3.6–8.0) and 2016 (48.9%) observations were from female subjects. The distribution of observations from children with and without signs of illness by age and RR are presented in Supplementary Table 1 and Supplementary Fig. 1.

### Predictors of respiratory rate among community children without signs of illness

The prevalence of stunting (1–14 years of age) was 21.1% (95% CI 19.4–22.9%), the prevalence of underweight (1–10 years of age) was 22.6% (95% CI 20.6–24.6%) and prevalence of wasting (1 to < 5 years of age) was 8.0% (95% CI 5.9–10.6%). Females contributed 1037 (47.7%) observations in the 3 cross-sectional surveys. The distribution of observations over age and RR measurements are presented in Supplementary Fig. 1A and C, and Table 1.

In a pooled adjusted mixed-effects linear regression (n = 800 children, contributing 12,215 observations); age (non-linear effect), stunting, minimum daily temperature (a proxy for ambient temperature), and time of day of data collection were associated with respiratory rate (Table 2). On average, stunting was associated with a 0.84 breaths/min (95% CI 0.40–1.28, *p* < 0.001) increase in RR. A 1 °C increase in minimum daily temperature was associated with a 0.38 breaths/min (95% CI 0.33–0.42, *p* < 0.001) increase in RR. In addition, there was a nonlinear relationship between RR rate measurements and the time of the day when the measurements were taken (Table 2). On average RR measurements increased from 0700 to ~ 1500 h after which the measurements declined towards 2000 h (*p* < 0.001). While rainfall was significantly associated with RR in the univariable model, it was not significant in multivariable models that included minimum temperature. No evidence of being female or enrolled in the intervention group was associated with RR in the adjusted regression models (Table 2). As observed in the GAMLSS model and consistent with previous studies^18,19^, older children had lower RR measurements in both the unadjusted and the adjusted regression models (Table 2). That is, compared to children aged 1-to-2-year-old; children aged 2-to-3-years, 3-to-4 years, 4-to-6 years, 6-to-8 years, and 8-to-13 years old had RRs that were 0.38, 2.15, 5.14, 9.56, and 10.64 breaths/min lower respectively (Table 2).

**Table 2:** Predictors of respiratory rate.

Covariate	Model 1: Univariable Analysis (n = 12,215)			Model 2: Multivariable Analysis (n = 12,215)		
	Change in RR	95% CI	*p* value	Change in RR	95% CI	*p* value
Age (ref, 1 to < 2 years)	ref			ref		
2 to < 3 years	− 0.18	− 1.87 to 1.50	0.831	− 0.38	− 2.22 to 1.48	0.698
3 to < 4 years	− 1.77	− 3.45 to − 0.08	0.041	− 2.15	− 4.04 to − 0.27	0.025
4 to < 6 years	− 4.93	− 6.49 to − 3.37	< 0.001	− 5.14	− 6.93 to − 3.35	< 0.001
6 to < 8 years	− 9.15	− 10.65 to − 7.65	< 0.001	− 9.56	− 11.32 to − 7.81	< 0.001
8 to < 13 years	− 10.12	− 11.61 to − 8.62	< 0.001	− 10.64	− 12.39 to − 8.90	< 0.001
Sex (ref, Male)	ref			ref		
Female	0.11	− 0.50 to 0.72	0.720	0.21	− 0.12 to 0.54	0.213
Randomization (ref, Control)	ref			ref		
Treatment	0.45	− 0.15 to 1.06	0.142	0.19	− 0.14 to 0.52	0.258
Time when RR measurements were taken (ref, 07:00–09:00 h)^i^	ref			ref		
09:00–11:00 h	0.36	− 0.07 to 0.78	0.099	0.36	0.06– 0.65	0.017
11:00–13:00 h	0.68	0.15–1.20	0.011	0.78	0.46–1.10	< 0.001
13:00–15:00 h	1.09	0.49–1.69	< 0.001	1.02	0.61–1.42	< 0.001
15:00–17:00 h	0.34	− 0.19 to 0.87	0.208	0.26	− 0.12 to 0.64	0.183
17:00–20:00 h	− 0.15	− 0.76 to 0.46	0.629	0.51	0.07 to 0.94	0.023
**Rainfall** (mm) on the day of data collection^ii^	0.03	0.02–0.04	< 0.001	0.001	− 0.01 to 0.01	0.935
**Minimum daily temp** (^o^C)	0.31	0.26–0.36	< 0.001	0.38	0.33 to 0.42	< 0.001
Stunting (ref, normal)^iii^	Ref					
Stunted	2.46	1.66–3.27	< 0.001	0.84	0.40 to 1.28	< 0.001
Wasting (ref, normal)^iii^	ref					
Wasted	− 0.74	− 2.27 to 0.80	0.347			
Underweight (ref, normal)^iii^	ref					
Underweight	0.47	− 0.24 to 1.18	0.191			

Model 1 presents results of the univariable mixed-effect linear regression model and Model 2 presents results from the final multivariable mixed-effect linear regression model. Sex and the randomization group have been included as potential confounders in the multivariable model.

^i^Time is defined as the time when the respiratory rate measurement was taken and was restricted to between 07:00 and 20:00 h respectively.

^ii^Rainfall (mm) and ambient temperature on the day when RR measurements were taken were retrospectively extracted from the meteorological department in Basse Santa Su town and linked to the RR dataset.

^iii^Stunting was defined as the height/length for age z-score < − 2 (HAZ/LAZ < − 2), wasting was defined as the weight for height z-score < − 2 (WHZ < − 2), and underweight was defined as the weight for age < − 2 (WAZ < − 2). The 2006 and 2007 WHO algorithm estimates HAZ/LAZ for children 6 months to 14 years, WAZ for children under 10 years, and WHZ for children under 5 years.

### Intra-observer variability in respiratory rate measurement

**Fig. 3:**
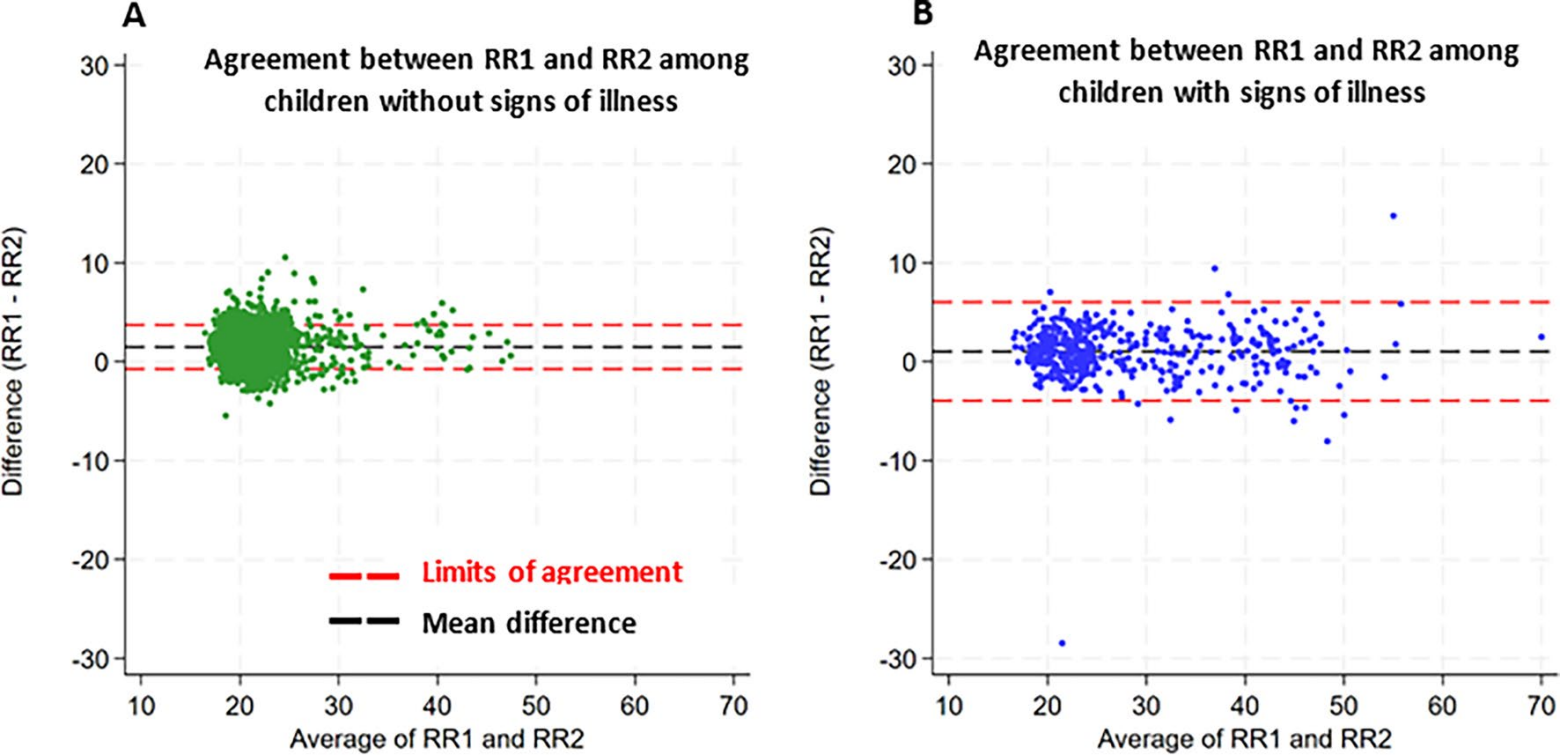
**Bland–Altman plots for RR measurements.** Panel A displays data from children without signs of illness whilst panel B displays data from children with signs of illness. The black dashed line represents the level of bias while the red dashed lines display the limits of agreement. RR1 is the first RRs measurement while RR2 is the second RRs measurement taken in that order. The mean difference in RR measurements was 1.49 breaths/min (limits of agreement: -0.74 to 3.71) for children without signs of illness and 1.04 breaths/min (limits of agreement: -3.94 to 6.02) for children with signs of illness. Data points are jittered for clarity.

A total of 2311 paired measurements were used in the reliability assessment. 1,159 (50.2%) of the paired measurements were from female subjects and 460 (19.9%) were from children with signs of illness. Overall, the ICC for RR among all children with paired measurements (age 1–13 years) was 0.93 (95% CI 0.93–0.94) whilst the ICC for children without signs of illness was 0.84 (95% CI 0.83–0.86). Among children aged less than 5 years, the ICC was 0.94 (95% CI 0.92–0.96) while the estimate for children above 5 years and < 14 years was 0.83 (95% CI 0.82–0.84). The Bland–Altman plots of intra-observer reliability in RR measurement are presented in Fig. 3A and B. The mean difference between paired RR measurements was small in both children with (1.49 breaths/min [limits of agreement: − 0.74 to 3.71]) and without (1.04 breaths/min [limits of agreement: − 3.94 to 6.02]) signs of illness (Fig. 3A and B). However, the variability between the differences in paired RR measurements among sick children (SD = 2.54) was higher than among children without (SD = 1.13) signs of illness (one-way ANOVA, *p* < 0.001).

## Discussion

The WHO definition of tachypnoea does not address in any detail children above 5 years of age^28^. To construct reference charts for children aged 1 to 13 years we used a longitudinal dataset of RR measurements derived from a large cohort of rural Gambian children enrolled in a trial investigating the impact of improved housing on the incidence of malaria^20,21^. These data were linked to potential environmental exposures, demographic factors, and three cross-sectional surveys on participant’s nutritional status. RR measurements were done to monitor pneumonia, a potential serious adverse effect, since there was concern that the housing modifications in the intervention arm would increase respiratory disease—however, this was shown not to be the case^20,21^. The constructed RR-for-age reference chart among children without signs of illness decreased with increasing age and is consistent with previous findings^17–19^.

On average, stunting was associated with a small increase in RR consistent with a recent multicounty pneumonia trial^29^ in which stunting was associated with a RR of 1 breath/min higher at hospital admission. Whereas the multicounty trial assessed children presenting with signs of pneumonia^29^, our study examined RR measurements among mainly healthy children in the community. Stunted children have been reported to have lower ventilatory reserve than children with normal height for age^30^. Stunting has been associated with reduced lung function resulting from impaired lung development^31–33^ while chronic undernutrition predisposes children to severe illnesses such as pneumonia that can potentially lead to long-term effects on lung development^32–35^. Therefore, the relatively higher RRs observed among stunted children may be compensatory for inadequate ventilation. Our results, however, contrast with a previous study conducted in The Gambia that documented relatively lower RR measurements among stunted children with signs of pneumonia^36^ thus highlighting the need for further research to confirm these findings.

Higher ambient temperature was associated with increased RR measurements consistent with previous studies in both human and animal models^37^. The higher RR observed at higher ambient temperatures may be a homeostatic mechanism by which the body maintains its normal temperature^37^. On average, RR measurements taken in the afternoon were higher compared to those taken in the morning or in the evening. Higher RR in the afternoon may be a result of the cumulative increase in the child’s activity during the day. Increased activity (exercise) has been associated with an increase in oxygen requirements leading to an increase in RR^38^. Another potential explanation is that ambient temperature increases towards the afternoon and may thus confound the time when measurements were taken. However, the precise ambient temperature when RR measurements were taken was not available for these analyses.

Our study has important strengths. First, few studies provide a comprehensive assessment of the factors associated with RR using a large cohort of children who were actively followed up to document their clinical disease status, nutritional status, and other potential environmental risk factors. Second, the RR measurements were taken from children drawn from randomly selected households in the community and therefore the findings are generalizable to the community. In contrast, most RR studies have relied on cross-sectional studies where data are collected in hospital or daycare centers and therefore difficult to generalize to the community. Third, although the repeat RR measurements were only taken among children whose initial measurements were considered raised for age, the intra-observer reliability index (ICC) in both the overall and subgroup analyses was > 0.8 suggesting good to excellent reliability^39^. In addition, we did not find any systematic differences between the repeat measurements as shown in the Bland Altman plot (Fig. 3A and B). The variability between the RR repeat measurements was higher among children with signs of illness than among children without signs of illness (*p* < 0.001), highlighting a potential limitation of utilizing a single RR measurement for the classification of pneumonia and other respiratory illnesses.

Our study has some limitations. We did not record whether the child was asleep or awake when the RR measurements were taken. Previous studies have shown that RR measurements taken when children are asleep are lower than those taken when children are awake^18^. However, measurements were only taken when the child was calm. In addition, our data were collected from rural Gambia, a country situated less than 60m above sea level with high seasonal temperatures. Therefore, the interpretation of our results may be limited to areas with similar geographical and climatic characteristics^17^.

In conclusion, the current WHO tachypnea guidelines do not address in any detail children above 5 years of age^28^. We constructed centile charts using data collected from a low altitude area in The Gambia and propose a cutoff of > 26 breaths/min for raised RR among children > 5 years and below 13 years of age. We demonstrate that age, time of data collection, nutritional status, and ambient temperature were associated with RRs. However, the small change in RR associated with nutritional status, time of day when RR measurements were taken and ambient temperature does not warrant a change in the current WHO tachypnea guidelines for children > 1 year and < 5 years of age. Consistent with a previous study^17^, we note that the WHO definitions for fast breathing in children under 5 years may misclassify vulnerable children with acute respiratory signs, leading to inappropriate management and misallocation of scarce resources like antibiotics and oxygen. The finding that RR differences between paired repeat measurements were more variable among children with signs of illness raises concerns over the reliability of a single RR measurement among children with signs of illness—a population needing accurate diagnosis to enable targeted interventions with lifesaving treatment.

### Supplementary Information

Supplementary Information.

## Data Availability

Access to clinical data requires a formal application to the Scientific Coordinating Committee of the Medical Research Council Unit The Gambia (MRCG) and the Joint Gambian Government’s and MRCG Ethics Committee in The Gambia at https://www.mrc.gm/scientific-coordinating-committee/
