# Supplementary material for: Respiratory rates among rural Gambian children: a community-based cohort study
Source: Sci Rep. 2024 Sep 2;14:20354. doi: 10.1038/s41598-024-70796-7 (PMC11369163; doi:10.1038/s41598-024-70796-7)
Supplement: Supplementary file 1 — Supplementary Information. [file 41598_2024_70796_MOESM1_ESM.docx]

**
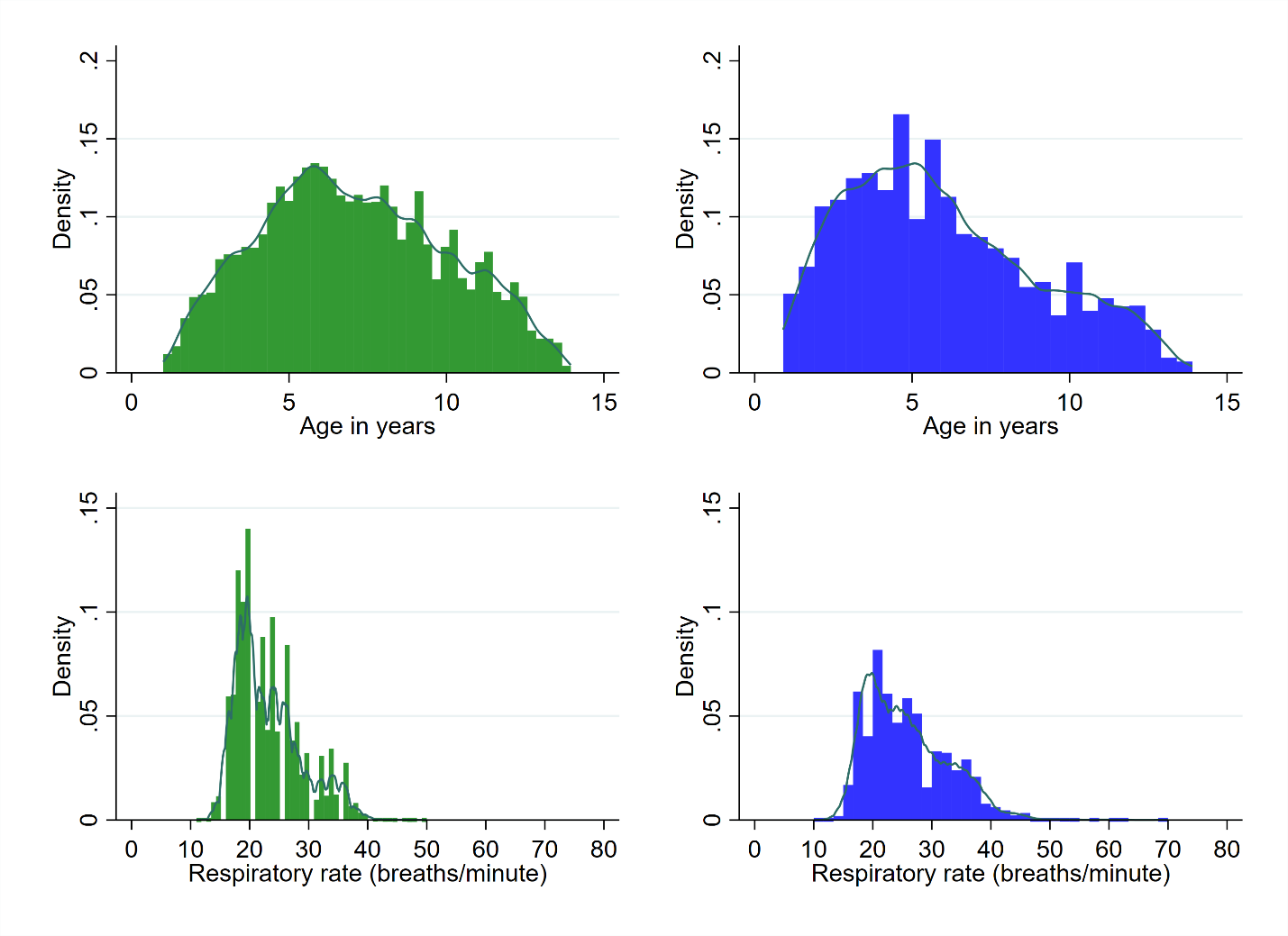
**

**Children with signs of illness-**

**Children without signs of illness-**

**Children with signs of illness-**

**D-**

**C-**

**Children without signs of illness-**

**A-**

**B-**

**Supplementary Figure 1: Histograms of the distribution of study participants by age and respiratory rate measurements.** Panels A and B show the distribution of age among children without and children with signs of illness respectively and panels C and D show the distribution of respiratory rate among children without and children with signs of illness respectively.

**Supplementary Table 1:** Summary of children with signs of illness, their corresponding observations, and median respiratory rate segregated by age

|  | Children with signs of illness | | | Children without signs of illness | | |
| --- | --- | --- | --- | --- | --- | --- |
| **Age in Years** | **Number of observations (%)** | **Number of children** | **Median RR (IQR)** | **Observations** | **Number of**  **Children** | **Median (IQR)** |
| 1 to <1.5 | 60 (13) | 16 | 35 (32 - 39) | 410 | 20 | 32 (28 - 35) |
| 1.5 to <2.5 | 407 (13) | 77 | 32 (29 - 36) | 2609 | 89 | 32 (28 - 35) |
| 2.5 to <3.5 | 492 (10) | 113 | 30 (27 - 34) | 4308 | 142 | 30 (26 - 34) |
| 3.5 to <4.5 | 514 (9) | 136 | 28 (25 - 34) | 5273 | 177 | 28 (24 - 33) |
| 4.5 to <5.5 | 566 (7) | 156 | 26 (22 - 30) | 7180 | 218 | 25 (21 - 30) |
| 5.5 to <6.5 | 510 (6) | 153 | 22 (19 - 24) | 7875 | 237 | 21 (19 - 25) |
| 6.5 to <7.5 | 369 (5) | 133 | 20 (19 - 24) | 6753 | 209 | 20 (19 - 24) |
| 7.5 to <8.5 | 315 (5) | 116 | 20 (18 - 24) | 6593 | 193 | 20 (18 - 23) |
| 8.5 to <9.5 | 227 (4) | 91 | 20 (19 - 24) | 5821 | 167 | 20 (18 - 24) |
| 9.5 to <10.5 | 231 (5) | 84 | 20 (18 - 23) | 4600 | 142 | 19 (18 - 22) |
| 10.5 to <11.5 | 176 (4) | 63 | 19 (18 - 21) | 3881 | 118 | 20 (18 - 22) |
| 11.5 to <12.5 | 179 (5) | 66 | 20 (19 - 23) | 3092 | 92 | 19 (18 - 22) |
| 12.5 to <13.5 | 67 (4) | 26 | 20 (18 - 22) | 1478 | 49 | 19 (17 - 22) |
